# Radiofrequency ablation versus hybrid argon plasma coagulation in Barrett’s esophagus: a prospective randomised trial

**DOI:** 10.1007/s00464-023-10313-5

**Published:** 2023-08-21

**Authors:** Mate Knabe, Jens Wetzka, Lukas Welsch, Johannes Richl, Florian Michael, Sandra Blößer, Myriam Heilani, Holger Kronsbein, Andrea May

**Affiliations:** 1 Department of Gastroenterology, Goethe University Frankfurt, University Hospital, Frankfurt am Main, Germany; 2 Department of Gastroenterology, Sana Klinikum GmbH Offenbach, Offenbach, Germany; 3 Department of Medicine I, Asklepios Paulinen Klinik Wiesbaden, Wiesbaden, Germany; 4 Department of Gastroenterology, Facility Bielefeld-Bethel, University Hospital Ostwestfalen-Lippe, Bielefeld, Germany

**Keywords:** Barrett, Radiofrequency ablation, Hybrid argon-plasma-coagulation

## Abstract

**Introduction:**

Radiofrequency ablation (RFA) and hybrid argon plasma coagulation (H-APC) are established thermal ablation techniques for eradicating Barrett’s esophagus after endoscopic resection. This study aimed to compare RFA with H-APC in relation to safety, effectiveness and eradication rates.

**Methods:**

After endoscopic resection, patients were randomly assigned to H-APC or RFA. A simplified H-APC technique was applied at 60 W. RFA was used with a 90° focal catheter and a simplified protocol of 12 J/cm^2^ × 3 or with a Halo 360° balloon and 10 J/cm^2^/cleaning/10 J/cm^2^. Eradication rates and adverse events were recorded. Patients received follow-up examinations after 3, 6, 12 and 24 months.

**Results:**

One hundred and one patients were finally included in the study (RFA *N* = 47, H-APC *N* = 54). The median follow-up period for short-term was 6.0 (CI 5.4–6.9) months and for long term 21 (CI 19.2.5–22.7) months. In total 211 ablations were performed. The eradication rates after long-term follow-up were 74.2% in the RFA group and 82.9% in the H-APC group. Post-interventional pain was significantly greater in the RFA group, with a mean score of 4.56/10 and duration of 7.54 days, in comparison with a mean score of 2.07/10 over 3.59 days in the H-APC group. Stenoses requiring intervention were noted in 3.7% of patients in the H-APC arm and 14.9% of those in the RFA arm.

**Conclusions:**

Both ablation techniques have good results in relation to the eradication rate, with a slightly better outcome in the H-APC group. The severity and duration of pain were significantly greater in the RFA group.

Thermal ablation has become established as the first-choice treatment after endoscopic resection of oesophageal neoplasia [1–3]. This approach has been confirmed in a large number of different retrospective and prospective trials.

Argon plasma coagulation (APC) was one of the first techniques that was used in this therapeutic field, with low complication rates [4]. In the APE study, ablation with APC was investigated in a randomised trial with a long-term follow-up period. The study reported a decrease in recurrent neoplasia from 36.7 to 3% in comparison with surveillance alone [5]. Radiofrequency ablation (RFA) was developed as an alternative method, with a low disease progression rate and a decreasing risk for neoplasia [6]. The short-term and long-term outcomes with both techniques show excellent results in relation to rates of eradication of Barrett’s esophagus, but several studies have reported a wide range of success rates, from 74 to 98% [4, 6–8]. The two therapeutic approaches have similar complications and drawbacks, as they are both based on thermal destruction of the Barrett’s mucosa. These adverse events mainly consist of acute bleeding, rare cases of perforation, and the development of post-treatment strictures. Stricture rates vary depending on the amount of energy applied and the size of the ablated area.

The standard RFA protocol includes a phase in which debris is cleaned from the surface and two treatments are applied at 15 J/cm^2^ (2 × 15 J/cm^2^ − cleaning − 2 × 15 J/cm^2^) [9]. As cleaning debris from the surface is time-consuming, there have been continuing efforts to develop a more simplified protocol. One randomised trial reported results that were not inferior to the standard regimen with regard to eradication rates, but the stricture rates increased to 11% [10, 11]. Maintaining the simplified protocol but lowering the energy to 12 J/cm^2^ appeared to be highly effective, with acceptable complication rates [12].

Complication rates after standard APC are comparably high (9.8%) [4]. Hybrid APC (H-APC) was developed to avoid post-treatment strictures. The procedure involves a saline injection into the submucosal tissue to avoid thermal injury to the deeper layers and muscularis. The first pilot study of the technique showed promising results, with complete macroscopic remission in 96% of cases and a stricture rate of 2% [13]. A large prospective multicentre trial confirmed good eradication rates (88.4%), with a promisingly low complication rate (6.1%) [14]. The recommended H-APC protocol involves a cleaning phase after 60 W APC (60 W − cleaning − 50 W). According to the present authors’ assessment and experience in the multicentre trial, a simplified protocol with 60 W H-APC without cleaning should be effective and safe.

There has only been one larger randomised controlled trial comparing RFA and APC, and there have been no trials so far comparing H-APC with RFA [15]. The present study investigated RFA using the simplified RFA protocol and a simplified H-APC protocol (Fig. 1).

**Fig. 1:**
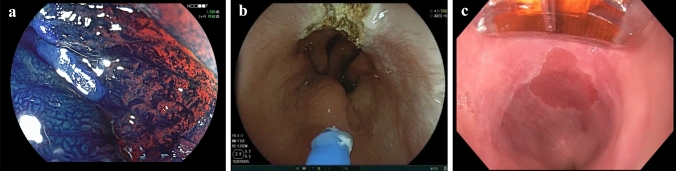
**a** Chromoendoscopy in combination with zoom endoscopy shows the margin of an early adenocarcinoma and non-neoplastic Barrett’s mucosa. **b** Hybrid-APC in a non-neoplastic Barrett at the 12 o’clock position. **c** RFA using a 90° focal catheter for ablation

## Material and methods

The study is a randomized trial and was conducted prospectively in a tertiary care centre in Germany (12/2017–05/2022). All of the endoscopists involved had sufficient experience in interventional Barrett’s esophagus therapy (each having treated over 100 patients with neoplastic Barrett’s esophagus). The study protocol was approved by the General Medical Council of the state of Hesse (FF100/2017) and registered in the German trial registry (DRKS-ID: DRKS00013026).

### Inclusion criteria

- Patients aged > 18 and < 99 with eradication of neoplastic Barrett’s esophagus following curative endoscopic resection of visible lesions (maximum T1sm1, G1/2, L0 V0 R0 basal) with planned complete Barrett’s esophagus eradication or primary ablation for low-grade intraepithelial neoplasia (LGIN) or macroscopically invisible high-grade intraepithelial neoplasia (HGIN).
- Barrett’s esophagus length M ≥ 1 cm (Prague classification).
- Informed consent.

### Exclusion criteria

- Patients with Barrett’s esophagus with no history of neoplasia.
- Patients in whom complete Barrett’s esophagus eradication was not planned.
- Previous ablation therapy.
- Patients with high-grade strictures after endoscopic resection who were not suitable for multiple dilation sessions.
- Patients with other cancers non-curatively treated.
- Severe comorbidity and life expectancy of < 1 year.
- Clotting disorders and oesophageal varices.
- Pregnancy.
- Lack of written consent.

### Study procedures

The *therapeutic aim* in these patients was to completely eradicate all neoplastic Barrett’s epithelium plus any remaining normal Barrett’s epithelium. The initial work-up with high-resolution endoscopy was performed using staining (e.g., with acetic acid) to identify and biopsy visible lesions.

*Endoscopic resection* was performed using a suck-and-cut technique. Endoscopic submucosal dissection was not performed in this group of patients.

*Argon plasma coagulation* was performed using the Hybrid-APC probe (Erbe, Tübingen, Germany) after prior injection with saline, using the same method described previously [13]. Ablation at 60 W was used as a standard. Cleaning of the mucosa with a transparent cap and another treatment at 50 W were not implemented in this study. At each follow-up examination, the distal esophagus was checked for residual islands of Barrett’s mucosa, which were then treated. In one of the later sessions, an attempt was made to ablate the oesophagogastric junction area about 1 cm into the cardia in a circumferential fashion. After therapy, proton-pump inhibitors were administered at 3 × 40 mg for 3 weeks, followed by 2 × 40 mg. The sessions were continued until the macroscopic impression at endoscopy suggested complete eradication, which was confirmed by biopsy. Treatment was continued up to a maximum of five ablation sessions, after which the case was counted as a failure of the initial treatment series. If significant neoplasia (re)appeared during the course of ablations, (i.e., visible lesions, histology, or more), the therapy was switched to endoscopic resection.

Radiofrequency ablation was performed using simplified protocol of 3 × 12 J/cm^2^ with focal 90° catheter or Balloon using 2 × 10 J/cm^2^ (Ablation-Clean-Ablation).

The study was divided into two stages. During ablation stage all patients received ablation at intervals of 8–12 weeks, until total Barrett’s eradication was confirmed or the maximal allowed number of five ablations were reached. This was followed by the surveillance phase with defined follow-up endoscopies.

All follow-up endoscopies were performed with chromoendoscopy and additional four quadrant biopsies. One follow-up endoscopy with negative biopsies (neo-Z line) was counted as *evidence of therapeutic success* of the Barrett’s eradication therapy. Short-term follow-up examinations were performed at 3 and 6 months after the last ablation. This was followed by long-term follow-up between 12 and 24 months after the last ablation. If there was a suspicion of Barrett’s epithelium at endoscopy and this was confirmed by a positive biopsy, or if biopsy alone was positive for intestinal metaplasia, this was counted as a recurrence, and therapy continued at the discretion of the endoscopist.

The *endoscopic definition* of Barrett’s esophagus initially used the Prague classification, and for the assessment of residual/recurrent Barrett’s, a minimum tongue of 1 cm (≥ Prague C0M1) after endoscopic resection was required for inclusion in this trial. Histological evaluation was carried out by a local highly experienced gastrointestinal pathologist. Barrett’s esophagus was diagnosed whenever specialised intestinal metaplasia (with goblet cells) was present. If LGIN or HGIN was observed, a second pathologist was involved. Histological diagnosis was always regarded as the gold standard—for example, in cases with normal endoscopic findings but Barrett’s mucosa in the histology.

Adverse events were documented during hospital stays and at the next check-up or treatment appointment. These included significant pain, fever, bleeding requiring interventions, perforation, or other findings. During each subsequent visit, patients were asked about dysphagia, odynophagia, or any other chronic oesophageal symptoms. The severity and duration of pain were recorded by interviewing the patients before discharge and at each appointment during the course of the study. Pain severity was recorded by visual scale from 1 = no pain to 10 = extensive pain. All patients were asked to protocol the duration of pain after discharge until pain has resolved.

Severe adverse events were defined as those requiring extra interventions, such as closure of perforations, additional interventions beyond APC for bleeding during the initial treatment and events requiring repeat interventions such as repeated endoscopy, surgery, intensive-care unit admission and/or prolongation of the hospital stay.

### Outcomes

The *primary outcome* was the rate of complete eradication of Barrett’s esophagus (neoplastic and normal Barrett’s esophagus), assessed by negative biopsy during a short-term follow-up endoscopy (up to 6 months).

*Secondary outcomes* included the rate of recurrence-free survival during the long-term follow-up period of 1–2 years; the number of ablation sessions; adverse events during ablation therapy, including measures required (e.g., dilation of strictures); and pain severity and pain duration after treatment.

### Statistical analysis

Descriptive analyses were carried out using IBM SPSS Statistics for Windows, version 27. Continuous variables were described using mean. Categorial variables were described by percentage or frequencies. A test for normal distribution of the present data was performed by Kolmogorov–Smirnov test. Differences between the two groups were analysed by *T*-Test or Mann–Whitney *U* Test.

Sample size calculation: The eradication rate with RFA ranges from 81 to 100%, with a mean of 90%. It was assumed that the rate of complete eradication with hybrid APC may be at most 10% poorer than that of RFA, so that equivalence between RFA and hybrid APC can be demonstrated using a non-inferiority design.

- RFA: p0 = 0.90.
- Hybrid APC: p1 = 0.8.
- *α* = 5%, power = 80%.

This sample size calculation results in *N* > 157 patients per group.

### Randomization

Randomization was done digitally for all consecutive patients who met the inclusion criteria. Randomization sequence was specified before first intervention. All patients had to sign informed consent and intervention was only performed by study physicians.

## Results

A total of 110 patients (age 42–82, mean 64.6 years) were randomized in this prospective trial. Nine patients had to be excluded before they completed all necessary ablations. Finally, 101 patients were included to this trial (Table 1). Patients were randomly assigned to receive radiofrequency ablation (*N* = 47) or hybrid APC (*N* = 54). There were no significant differences between the group with regard to the length of Barrett’s mucosa before the start of ablation therapy (Table 2). Between September 2017 and November 2021, 211 ablations were performed.

**Table 1:** Histological results before inclusion in the study

Histology	N
LGIN	15
HGIN	16
Mucosal cancer	64
Submucosal cancer	6

*HGIN* high-grade intraepithelial neoplasia; *LGIN* low-grade intraepithelial neoplasia

**Table 2:** Main outcomes during the treatment phase and endoscopic surveillance

	RFA (*N* = 47)Mean (min.–max.)	H-APC (*N* = 54) Mean (min.–max.)	Study population (*N* = 101)	Mann–Whitney test *p* = 0.05
Barrett’s length				
Circumferential	2.60 cm (CI 1.55–3.65)	1.46 cm (CI 0.70–2.23)	2.68 cm (CI 1.99–3.37)	0.08
Maximum	4.72 cm (3.59–5.86)	4.33 cm (CI 3.41–5.26)	4.92 cm (CI 4.23–5.61)	0.877
Ablations	2.09 (CI 1.74–2.43)	2.19 (CI 1.89–2.48)	2.14 (CI 1.91–2.36)	0.513
	Total: 98	Total: 113	Total: 211	
Ablation length per session	4.51 cm (CI 3.83–5.20)	3.24 cm (CI 2.73–3.75)	3.83 cm (CI 3.40–4.27)	0.003
Circumferential ablation	Table Nr 3	Table Nr 3	Table Nr.3	
Ablation time per session	7.72 min (CI 6.76–8.68)	7.02 min (CI 6.28–7.77)	7.34 min (CI 6.75–7.94)	0.90
Adverse events				
Total	2.1%	0.8%	1.4%	
Fever	0.0%	0.8%	0.5%	
Major bleeding	1.0%	0.0%	0.5%	
Minor bleeding	1.0%	0.0%	0.5%	
Stenosis	14.9%	3.7%	8.9%	0.05
Pain (scale 1–10)	4.56 (CI 3.70–5.42)	2.07 (CI 1.44–2.69)	3.11 (CI 2.50–3.72)	*p* < 0.001
Pain duration (days)	7.54 (CI 4.62–10.46)	3.59 (CI 1.75–5.43)	5.24 (CI 3.58–6.89)	*p* < 0.001
Follow-up study population *N* = 84				
Short-term follow up (median 6.0 CI 5.4–6.9)	31	43	74	
Residual Barrett’s	11 (35.5%)	9 (20.9%)	17 (20.2%)	0.129
Long-term follow up (median 21.0 CI 19.2–22.7)	31	41	72	
Residual Barrett’s	8 (25.8%)	7 (17.1%)	15 (17.9%)	0.07

*RFA* radiofrequency ablation; *H-APC* hybrid argon plasma coagulation; *SD* standard ablation

### Hybrid-APC

Ablation with H-APC involves prior injection of saline into the submucosal layer. The mean amount of saline injected into the submucosa was 9.19 mL (range 3–30 mL). Ablation was carried out at 60 W without cleaning and without a second ablation. The circumferential area and the length of the ablation was recorded for each intervention. Circumferential ablation was avoided in patients with long-segment Barrett’s esophagus, in order to prevent strictures. As therapy continued, the area with Barrett’s mucosa was reduced step by step. At the level of the gastro-oesophageal junction, H-APC was performed circumferentially. Table 2 provides an overview of ablation times and estimated areas.

### Radiofrequency ablation

Radiofrequency ablation was carried out using a 90° focal catheter or balloon. All ablations with the focal device used the simplified protocol (3 × 12 J/cm^2^). In 16.5% of all interventions, a balloon device was used with 10 J/cm^2^ − cleaning − 10 J/cm^2^. Using the focal device, circumferential ablation was only performed at the level of the gastro-oesophageal junction or with a balloon. Table 2 provides an overview of ablation times and estimated areas.

### Adverse events

Patients with post therapeutic stenosis after EMR (2.9% *N* = 3) were excluded from the final evaluation for any adverse events. The overall complication rate in the 211 ablations was low. There was only one case of acute bleeding with a need for endoscopic intervention. One patient developed fever after H-APC. One patient was admitted to hospital because of gastrointestinal hemorrhage. No perforations occurred in the present study population.

8.9% (9/101) were diagnosed with oesophageal stenoses and an indication for dilation after ablation. In the respective groups, relevant stenosis developed in 3.7% (2/54) of cases in the H-APC arm and in 14.9% (7/47) of those in the RFA arm. All of the stenoses were treated effectively using endoscopic dilation. To exclude the possibility that RFA balloon treatment might increase the complication rate, a subgroup analysis was performed. None of the patients who were treated with a 360° RFA balloon developed stenoses.

Patients were asked to record their pain levels by visual score from 1 to 10 and pain duration after discharge. This scores were recorded ad discharge and at follow-up endoscopy. Patients recorded higher pain levels after RFA, with a mean score of 4.56, than after H-APC, with a mean score of 2.07. The duration of pain was measured in days. Patients reported a longer period of pain after RFA, at 7.54 days, in comparison with H-APC, at 3.59 days.

### Follow-up

Seventeen patients were excluded from the follow-up analysis: One patients died not related to the study, another died due to bronchial carcinoma and thus did not receive any surveillance, and one was incorrectly given H-APC at the last intervention, although assigned to RFA. Due to pandemic or other reasons another 14 patients never showed up for surveillance endoscopy. Contact to patients were either not possible or control endoscopy was not desired.

All remaining patients (*N* = 84) received follow-up examinations, 74 with short-term follow-up (3–6 months) and 72 patients with long-term follow-up.

#### Short-term follow-up

In accordance with the protocol, follow-up examinations were planned 3 and 6 months after the last ablative therapy. Seventy-four patients (RFA: *N* = 31, HAPC: *N* = 43) received short-term follow-up examinations. This period was in median 6.0 months.

In the RFA and H-APC groups, Barrett’s metaplasia was diagnosed in 35.5% (11/31) and 20.9% (9/43) of the patients, respectively. Patients with macroscopically minimal residual Barrett’s at the neo-Z line, but negative findings at histology were counted as eradicated for Barrett.

In summary, the rates of Barrett’s eradication during the short-term follow-up were 64.5% (20/31) in the RFA arm and 79.1% (34/43) in the H-APC arm.

#### Long-term follow-up

Long-term follow-up was defined as an endoscopy examination more than 12 months after the last ablation.

Seventy-seven patients received long-term follow-up examinations after 12–24 months, with a median follow-up period of 21.0 months (CI 19.2–22.7 months). However, only 26.7% (23/86) of the study population patients followed the complete study as per protocol, which means surveillance 3, 6, 12 and 24 months after the last therapy.

After RFA, eight patients 25.8% (8/31) had histologically confirmed residual Barrett’s mucosa at the long-term follow-up examination, in comparison with 17.1% (7/41) with positive biopsy findings after H-APC. Ten patients with long-term endoscopy follow-up showed macroscopically minimal residual Barrett’s at the neo-Z line, but had negative histology findings. These patients were counted as eradicated for Barrett.

**Fig. 2:**
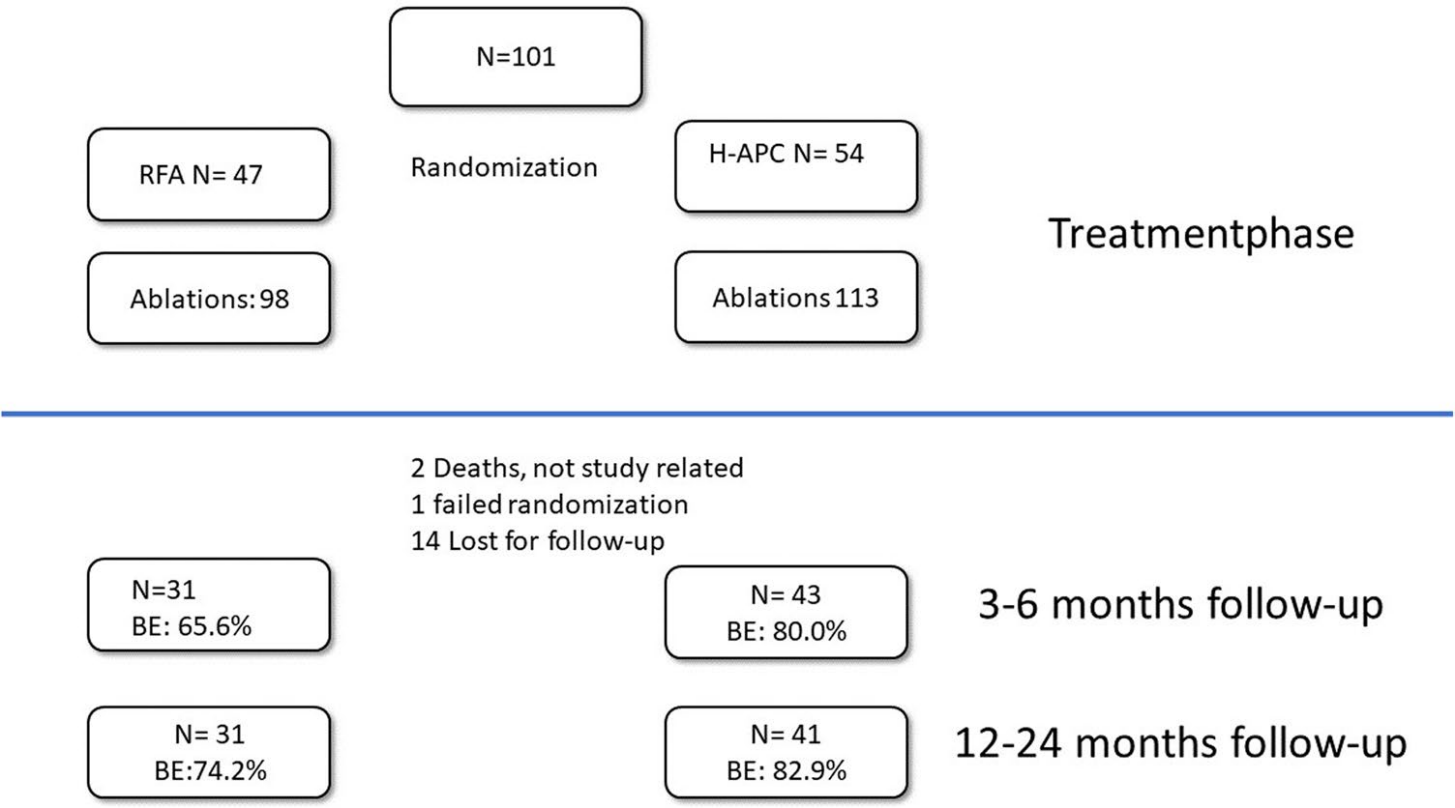
The flow chart shows treatments and follow-up examinations after randomisation for all patients. *BE* Barrett’s eradication; *H-APC* hybrid argon plasma coagulation; *RFA* radiofrequency ablation

In summary, the Barrett’s eradication rate during the long-term follow-up was 74.2% (23/31) in the RFA arm and 82.9% (34/41) in the H-APC arm (Fig. 2).

### Recurrent neoplasia

There was a low rate of recurrent neoplasia. Only one patient had a low-grade neoplasia 3 months after the last H-APC. The initial cancer had been graded T1a m1 G1 and was treated safely by endoscopic resection. All of the patients received histological assessments during surveillance, with the exception of two patients who only had macroscopic evaluations. One of these patients was receiving anticoagulation therapy, and the other patient only presented at the 1-year follow-up, without visible residual Barrett’s mucosa. A histological sample was not taken.

### Post-hoc analysis

Although without significant differences, the circumferential Barrett length was slightly increased in the RFA group compared to H-APC group (Table 3). We have performed different stratified analysis of eradication rate based on the lesion size to see if we can find a correlation. But all analysis remained without statistical significance. This result had perhaps changed when the full power of the study had been reached.

**Table 3:** Amount of circular ablation for Hybrid APC, RFA- 90 and Balloon device

Circular ablation per session								Chi-square test *p* < 0.05
	100%	75%	66%	50%	33%	25%	< 25%	
Hybrid APC	57	5	4	9	29	6	3	*p* = 0.06
RFA	60	5	4	15	13	1	0	
RFA-balloon	16	0	0	0	0	0	0	
RFA focal 90°	44	5	4	15	13	1	0	

## Discussion

Choosing the optimal ablation technique is likely to become increasingly important in the treatment of Barrett’s carcinoma in the future. Various methods have been emerging in recent years. The main points that speak for or against a method are ease of handling, the complication rate and the long-term results. Radiofrequency ablation is the method of first choice in most countries. It is regarded as being easy to learn, time-saving and effective. The traditional alternative is argon plasma coagulation. Both methods show good results, with moderate complication rates [13, 16]. In addition to these major techniques, there are a few emerging devices for ablative therapy that have not yet found their way into clinical practice. Cryotherapy shows a wide range of differences in efficacy and safety in the studies published so far, with eradication rates in most recent studies varying from 57 to 91% [17–19]. However, there is only one large prospective study such as those available for heat-based ablation [19].

Another nonthermal ablation technique using a through-the-scope device to resect nondysplastic Barrett’s mucosa is the EndoRotor [20]. In a pilot study, 14 patients were treated with the EndoRotor, but the rate of bleeding complications was around 37%. A large prospective trial is underway, but the results are pending. Both nonthermal ablation systems promise low stricture rates, but this has yet to be confirmed by larger prospective trials.

APC was the standard treatment until the introduction of RFA. Comparison of the two thermal ablation techniques is difficult, as RFA includes a balloon-based 360° device and focal devices with different lengths. In addition, the protocol for focal RFA has changed several times in recent years. The standard protocol, with 15 J/cm^2^ and an intermittent cleaning phase, has been reduced to a simplified protocol with the energy lowered to 12 J/cm^2^ and omission of cleaning [12]. This is the protocol mostly used in referral centres at the time the study was set up. Two fully published studies have so far used the simplified protocol with 3 × 12 J/cm^2^. However, there have been several oral presentations and an abstract, which are summarised in a published meta-analysis [12, 21]. The stricture rate using a simplified focal device at 3 × 12 J/cm^2^ was between 7.5 and 18% [22], with Barrett’s eradication rates of 60–91.7% [12, 21]. Only one large prospective, randomised study has investigated APC and RFA together. In the BRIDE study, 76 patients were randomly assigned to either conventional APC or RFA with a standard protocol. The results showed similar dysplasia clearance, safety and quality of life for RFA and APC, but there was a substantial difference in cost in favour of APC [15, 23]^.^

Hybrid APC, as a further development of conventional APC, shows good safety results and has sufficient data in the treatment of Barrett’s esophagus [13, 14, 24, 25]. Barrett’s eradication was satisfactory with RFA and H-APC. The Barrett’s eradication rates during the long-term follow-up were 74.2% for the RFA arm and 82.9% in the H-APC arm, without any significant differences.

There are several important points that have influenced the present trial. The difference of adverse events between the two arms was unacceptably high, with a stenosis rate of 14.9% in the RFA arm versus 3.7% in the H-APC arm. When the study was initially planned, the simplified protocol had good published data, with a stenosis rate of around 9% [12]. Since then, more data have become available and the stenosis rates have increased to 12.5–15% [21]. Therefore, the simplified protocol is no longer recommended by the company itself. On the other hand, a European multicentre trial using Hybrid-APC devices reported lower stenosis rates in 2021 that are almost identical to those in the present study (3.9%) [14]. This difference is probably explained by two major facts. The saline cushion prevents heat damage to the lower muscle layers and leads to decrease in stricture rates. Stricture development is based mainly on three facts. Applied energy to the surface, circumferential area and longitudinal ablation area. The energy was determined by the study protocol. The proportion of circumferential area per session is shown in Table 3. There was no significant difference comparing circumferential ablation between the two groups. In view of the significant difference in the ablation length between the two groups, that might be regarded as one explanation for the higher stenosis rate in the RFA arm. The difference is mainly due to two facts: 16.3% of all RFA patients were initially treated with a 360° balloon and a comparatively long ablation surface, but none of these patients developed stenoses. This is probably because the energy applied was only 10 J/cm^2^. Secondly, the 90° focal device has a minimum length of 2 cm, whereas only a length of 1 cm ablation is possible with H-APC. This factor was particularly evident in the last sessions, when there was only very little residual Barrett’s mucosa present. In addition, the patients in the RFA arm experienced pain for significantly longer periods (7.5 vs 3.6 days) and more intensively (4.6 vs 2.1) than those in the H-APC arm.

Most important weakness of the study is, that the calculated sample size was not reached. In respect to the present data and from ethical point of view, we believe that a continuation of the study on this basis is not give due to the large difference in adverse events between the various arms. However, the study is still the largest prospective randomized trial published so far. And the only published data comparing Hybrid-APC with RFA. With over 100 randomized patients it has relevant clinical impact. More over this, the variables used 2018 led to an initially over-calculated sample size as we have now much more scientific data on RFA simplified protocol and Hybrid-APC.

Another major limitation of this trial was the low rate of possible per-protocol analysis. Only 26.7% of all patients completed the full study protocol, with follow-up appointments after 3, 6, 12 and 24 months. This was largely due to a lack of follow-up endoscopies as a result of the Covid-19 pandemic. Particularly during the first wave of Covid-19, all surveillance endoscopies were postponed.

In conclusion, Hybrid-APC was used in this trial in a simplified form with only one ablation and without cleaning. This protocol is confirmed as safe, without any relevant drawbacks. It has rates of Barrett’s eradication similar to those with RFA, but the complication rate, and in particular the stricture rate, argue in favour of H-APC for thermal treatment in Barrett’s esophagus after endoscopic resection [26].

## Abbreviations

APC: Argon plasma coagulation
H-APC: Hybrid argon plasma coagulation
HGIN: High-grade intraepithelial neoplasia
LGIN: Low-grade intraepithelial neoplasia
RFA: Radiofrequency ablation
